# Alginate‐Based Encapsulation of *Pistacia lentiscus* (Mastic Gum) Extract and Its Application in Functional Kefir: Physicochemical, Antioxidant, and Textural Properties

**DOI:** 10.1002/fsn3.72262

**Published:** 2026-08-16

**Authors:** Özge Duygu Okur

**Affiliations:** ^1^ Department of Food Engineering, Faculty of Engineering Zonguldak Bulent Ecevit University Zonguldak Turkey

**Keywords:** alginate beads, bioactive compounds, fermented milk, phenolic compounds, plant extract

## Abstract

Bioactive compounds derived from natural sources are increasingly incorporated into functional dairy products; however, their limited stability during processing and storage may affect their functional performance. Encapsulation has been applied as a strategy to improve the stability and controlled delivery of sensitive bioactive compounds in food matrices. In this study, *Pistacia lentiscus* (mastic gum) extract was encapsulated using alginate and incorporated into kefir to evaluate the effects of free and encapsulated extract on physicochemical, antioxidant, and textural properties during refrigerated storage. The encapsulation efficiency of alginate beads was 78.15%, with a loading capacity of 15.30%. Kefir samples containing free or alginate‐encapsulated mastic gum extract at two concentrations were evaluated during 14 days of refrigerated storage. Total phenolic content and antioxidant activity ranged from 802.50 to 865.00 mg GAE/L and from 5.87 to 14.08 mM TE, respectively, depending on formulation and storage period. Encapsulated formulations exhibited phenolic content comparable to those containing free extract. Water‐holding capacity values ranged from 34.96% ± 0.20% to 47.10% ± 0.37%, while color and textural properties were influenced by formulation and storage time, with significant differences observed only in firmness among specific encapsulated formulations. These findings suggest that alginate encapsulation may contribute to the stabilization and controlled delivery of mastic gum bioactive compounds in kefir, supporting the development of fermented dairy products enriched with plant‐derived functional ingredients.

## Graphical abstract

*Pistacia lentiscus* (mastic gum) extract was obtained and encapsulated using sodium alginate by ionic gelation. The encapsulated extract was incorporated into kefir at different concentrations and compared with free extract and control kefir during refrigerated storage at 4°C for 14 days. Encapsulation efficiency, loading capacity, and mean bead diameter were determined, and the kefir samples were evaluated for total phenolic content, antioxidant activity, color properties, water‐holding capacity, texture, and microstructure by scanning electron microscopy. The results demonstrated enhanced antioxidant activity, improved retention of phenolic compounds, and better stability of selected physicochemical and textural properties during storage, supporting the potential of encapsulated *Pistacia lentiscus* extract for the development of functional kefir.

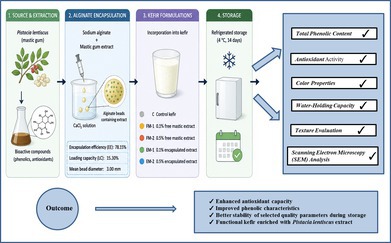

## 1 Introduction

Functional foods have attracted increasing scientific and commercial attention due to their potential to provide physiological benefits beyond basic nutritional requirements (Arshad et al. 2025; Balaji et al. 2026). Among fermented dairy products, kefir has gained particular interest as a functional beverage owing to its unique microbial diversity, naturally occurring bioactive metabolites, and potential health‐promoting properties. Kefir is a symbiotic fermented milk product produced by the metabolic activity of lactic acid bacteria and yeasts, resulting in the formation of bioactive compounds such as peptides, organic acids, exopolysaccharides, and other microbial metabolites. These compounds have been associated with beneficial effects, including modulation of gut microbiota, antioxidant activity, antimicrobial effects, and immune‐related functions (Vieira et al. 2021; Gruskiene et al. 2021; Marcìa‐Fuentes et al. 2026). However, the stability and functionality of bioactive compounds in fermented dairy matrices may be affected by processing conditions, storage, and interactions with food components, which may limit the effectiveness of functional ingredients incorporated into such products (Baars et al. 2023; Apalowo et al. 2024; Kurniawan et al. 2026).

*Pistacia lentiscus* L. (mastic gum) is a natural resin traditionally used for medicinal purposes and has recently gained attention as a potential source of functional bioactive compounds. Chemical characterization studies have demonstrated that mastic gum contains phenolic acids, flavonoids, terpenoids, and other phytochemicals responsible for its reported antioxidant, antimicrobial, and anti‐inflammatory activities (Soulaidopoulos et al. 2022; Boke Sarikahya et al. 2024; Kilic Buyukkurt et al. 2025; Labhar et al. 2025; El Allaoui et al. 2025). Due to its rich phytochemical profile, mastic gum extract represents a promising candidate for incorporation into functional food formulations. However, the direct application of plant‐derived extracts in food systems can be challenging because bioactive compounds may exhibit limited stability and may interact with food matrix components, potentially affecting their bioavailability and technological performance.

The incorporation of plant‐derived bioactive compounds into functional foods is often limited by their susceptibility to oxidation, light, temperature variations, pH changes, and interactions with other food components. These limitations may result in reduced bioactivity, poor bioavailability, and decreased effectiveness during processing and storage. Therefore, stabilization strategies based on protective carrier systems have gained increasing importance for maintaining the functional performance of bioactive compounds in food applications. Recent studies have highlighted that microencapsulation can act as a physical barrier between sensitive compounds and environmental stress factors, improving storage stability, controlled release behavior, and the applicability of natural bioactives in functional food formulations (Estevinho 2025; Abdel‐Aty et al. 2025; Barutçu Mazi et al. 2026; Kumari et al. 2026).

Among the available stabilization strategies, encapsulation has been widely investigated as an effective approach to improve the stability and controlled delivery of sensitive bioactive compounds in food applications. Encapsulation systems can protect phenolic compounds against environmental factors such as oxidation, pH variations, and storage‐related degradation, while also minimizing undesirable interactions with the surrounding food matrix (Obayomi et al. 2026). Among different encapsulating materials, alginate, a natural polysaccharide obtained from brown algae, has received considerable attention due to its biocompatibility, biodegradability, and ability to form stable gels through calcium‐mediated ionotropic gelation. Alginate‐based encapsulation has been reported to enhance the stability of phenolic compounds, improve probiotic survival, and provide controlled release properties in food matrices (Mohan et al. 2024; Jin and Wang 2026).

In fermented dairy systems, alginate encapsulation has mainly been explored for the protection of probiotic microorganisms and plant‐derived bioactive compounds. Previous studies have demonstrated that encapsulation may contribute to improved microbial viability, enhanced structural stability, and preservation of functional properties during storage and gastrointestinal conditions (Terpou et al. 2018; Yilmaz et al. 2020). Nevertheless, the encapsulation of mastic gum extract and its behavior within fermented dairy matrices remain insufficiently investigated. Particularly, limited information is available regarding how free and encapsulated mastic gum extracts influence the physicochemical properties, antioxidant capacity, and textural characteristics of kefir during storage.

The incorporation of mastic gum extract into kefir may involve several technological challenges, including the protection of phenolic compounds during fermentation, possible interactions between extract components and milk proteins, and changes in the structural properties of the final product. Therefore, understanding whether alginate‐based encapsulation can preserve the functional potential of mastic gum extract while maintaining the quality attributes of kefir is important for the development of innovative fermented dairy products.

Accordingly, this study aimed to encapsulate *Pistacia lentiscus* extract using alginate and evaluate the effects of free and encapsulated extracts on the physicochemical, antioxidant, and textural properties of functional kefir. This approach provides insight into the potential of alginate‐based encapsulation as a strategy for improving the stability and technological applicability of mastic gum‐derived bioactive compounds in fermented dairy systems.

## 2 Materials and Methods

### 2.1 Materials

Ultra‐high temperature (UHT) whole cow's milk was procured from a commercial dairy company located in İzmir, Turkey. Food‐grade powdered mastic gum, originating from Chios Island (Greece), was procured from a commercial supplier based in Ankara, Turkey. A commercially available freeze‐dried kefir starter culture was obtained from a manufacturer based in Poland. ABTS (2,2‐azinobis (3‐ethylbenzothiazoline‐6‐sulfonic acid) diammonium salt), Trolox (6‐hydroxy‐2,5,7,8‐tetramethylchroman‐2‐carboxylic acid), and gallic acid were obtained from Acros Organics (Morris Plains, NJ, USA). The Folin–Ciocalteu reagent was acquired from Merck (Darmstadt, Germany). Potassium persulfate (K_2_S_2_O_8_), sodium carbonate (Na_2_CO_3_), sodium alginate, sodium chloride (NaCl), and calcium chloride (CaCl_2_) were purchased from Sigma‐Aldrich (St. Louis, MO, USA). All chemicals and reagents employed throughout the study were of analytical grade.

### 2.2 Preparation of Mastic Gum ( *Pistacia lentiscus* ) Extract

Mastic gum extract was prepared by dispersing 3 g of powdered mastic gum in 150 mL of distilled water (1:50, w/v). The mixture was stirred at 400 rpm for 60 min at room temperature (25°C ± 1°C) to ensure complete dispersion and extraction of soluble components. After extraction, the suspension was allowed to settle and initially filtered through a coarse filter to remove insoluble residues, followed by filtration through Whatman No. 1 filter paper. The obtained aqueous extract was used immediately for subsequent encapsulation experiments.

### 2.3 Alginate Encapsulation of Mastic Gum Extract

**FIGURE 1:**
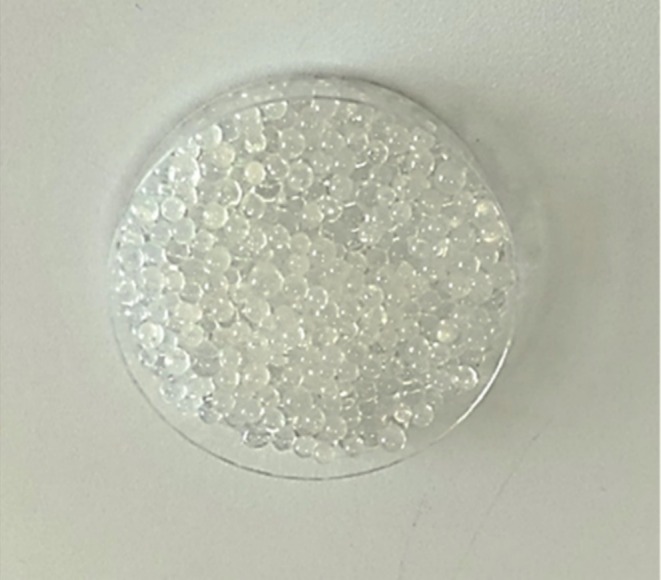
Alginate beads with mastic gum extract.

Sodium alginate (2 g) and NaCl (1 g) were dispersed in 100 mL of mastic gum extract under continuous stirring. The mixture was heated at 50°C for 3 min and homogenized for 2 min using a laboratory homogenizer (Homogenizer T 65, IKA, Staufen, Germany) to obtain a uniform polymer solution. The alginate–extract mixture was then loaded into a syringe equipped with a needle (21 G) and extruded dropwise into a continuously stirred (200 rpm) chilled calcium chloride solution (2%, w/v). The dropping distance was approximately 10 cm. Ionic gelation was allowed to proceed for 30 min to ensure complete bead formation. The formed alginate beads were collected by filtration, rinsed with distilled water to remove excess calcium ions and surface‐bound extract, and subsequently used directly in kefir formulations (Figure 1). The encapsulation procedure was performed according to Okur and Aboeldahab (2025) with minor modifications.

### 2.4 Encapsulation Efficiency, Loading Capacity and Size of Alginate Beads Containing Mastic Gum Extract

The encapsulation efficiency (EE) of beads containing mastic gum extract was determined based on their total phenolic content (TPC). Initially, the TPC of the extract solution used for encapsulation was measured using the Folin–Ciocalteu method and expressed as milligrams of gallic acid equivalents (GAE) per mL (Singleton and Rossi 1965). To determine surface phenolics (SP), 2 g of freshly prepared beads were gently rinsed with 10 mL of distilled water at room temperature for 1 min to remove phenolic compounds located on the bead surface. The washing solution was subsequently analyzed for TPC using the same method. For total phenolics (TP), another 2 g of beads were completely dissolved in a 2% (w/v) sodium citrate solution under gentle stirring for 10 min to release the encapsulated phenolic compounds, as previously reported for alginate microcapsules (Flamminii et al. 2021). The resulting solution was filtered prior to TPC determination. Encapsulation efficiency was calculated according to previously reported methods based on phenolic content differences (González Morales et al. 2025) using Equation (1):

(1) $$\mathrm{EE}\;\left(\%\right)=\left(\mathrm{TP}-\mathrm{SP}\right)/\mathrm{TP}\times 100$$

where TP represents the total phenolic content of the dissolved beads and SP denotes the surface phenolics obtained from the washing step. Two independent encapsulation batches were prepared for the two independent kefir production trials. All analyses were performed in duplicate within each batch.

The loading capacity (LC) of the alginate beads containing mastic gum extract was determined based on TPC (Singleton and Rossi 1965; Tolve et al. 2021). LC was calculated using the phenolic content retained within the beads after removing surface phenolics. The difference between TP and SP represents the amount of encapsulated phenolic compounds. The dry weight of the beads was determined after drying at 40°C until constant weight. LC (%) was calculated using Equation (2):

(2) $$\mathrm{LC}\;\left(\%\right)=\left(\mathrm{TP}-\mathrm{SP}\right)/W\times 100$$

where TP (mg GAE) is the total phenolic content of the dissolved beads, SP (mg GAE) is the surface phenolic content obtained after washing, and W (g) is the dry weight of the beads used in the analysis. All analyses were performed in duplicate for each of two independent experimental batches.

The diameter of 10 randomly selected freshly prepared (wet) capsules from each batch was measured using a digital caliper model 150 mm/6″ Digital Vernier Caliper (ElectroPeak, Friendswood, TX, USA), and the mean diameter ± standard deviation (SD) was calculated (Thomas Busani et al. 2020). These capsules were directly used in the kefir formulations without drying.

### 2.5 Scanning Electron Microscopy (SEM) Analysis

The morphology of alginate‐encapsulated mastic gum ( *Pistacia lentiscus* ) extract microcapsules was examined using a Quanta FEG 450 Scanning Electron Microscope (FEG SEM, Thermo Fisher Scientific, USA), as described by Yourdkhani et al. (2017). For this purpose, the microcapsules were first dried in an oven at 40°C for 24 h. Prior to imaging, the samples were coated with a thin layer of gold (~10 nm) to enhance conductivity. Imaging was performed under high vacuum mode at an accelerating voltage of 10 kV using a backscattered electron (BSE) detector. The working distance ranged from 12.2 to 12.6 mm, and micrographs were obtained at magnifications ranging from ×50 to ×10,000. The surface morphology, shape, and structural integrity of the microcapsules were evaluated.

### 2.6 Kefir Production

Kefir was produced according to the method described by Mudoor Sooresh et al. (2025) with slight modifications. The freeze‐dried kefir starter culture was first reactivated in skimmed milk and incubated at 25°C until the pH reached 4.6, after which it was stored at 4°C overnight. The approximate incubation time required to reach pH 4.6 was 16–18 h. UHT whole milk was heat‐treated at 85°C for 10 min and then cooled to 25°C. The treated milk was fortified with either free mastic gum extract (0.1% and 0.5%, w/v) or alginate‐encapsulated mastic gum microspheres at the same concentrations (0.1% and 0.5%, w/v). The samples were thoroughly mixed to ensure homogeneous distribution of the added ingredients. The fortified milk was inoculated with 3% (v/v) of the activated kefir culture and dispensed into 500 mL sterile glass jars. Fermentation was carried out at 25°C until the pH decreased to 4.6. After fermentation, kefir samples were stored under refrigerated conditions (4°C), and Day 1 was considered as the initial storage point after cooling. Analyses were performed on Days 1, 7, and 14 to evaluate the changes in physicochemical, antioxidant, and textural properties during storage. Five different kefir formulations were prepared, and their compositions are summarized in Table 1. Each formulation was produced in two independent kefir production trials conducted 1 week apart, and each production trial was considered an independent biological replicate. Samples from each production trial were stored and analyzed separately throughout the storage period. Within each biological replicate, analytical measurements were performed as technical replicates according to the respective analytical method, including duplicate measurements for pH, titratable acidity, total phenolic content, antioxidant activity, and water‐holding capacity, triplicate measurements for color, and four repeated measurements for texture analysis.

**TABLE 1:** Composition of the kefir formulations used in the study.

Formulation	Description
C	Control kefir
FM‐1	Kefir supplemented with 0.1% (w/v) free *Pistacia lentiscus* extract
FM‐2	Kefir supplemented with 0.5% (w/v) free *Pistacia lentiscus* extract
EM‐1	Kefir supplemented with 0.1% (w/v) alginate‐encapsulated *Pistacia lentiscus* extract
EM‐2	Kefir supplemented with 0.5% (w/v) alginate‐encapsulated *Pistacia lentiscus* extract

### 2.7 Characterization of Kefir

#### 2.7.1 Physicochemical Methods

Titratable acidity of the kefir samples was measured and expressed as lactic acid equivalents following the method described by Case et al. (1985). The pH of each sample was determined using a digital pH meter (Lab 860, Schott Instruments, Germany). Water‐holding capacity (WHC) was assessed according to the procedure outlined by Bielska et al. (2021). Briefly, 10 g of each kefir sample was centrifuged at 4100 × *g* for 30 min at 4°C. After centrifugation, the supernatant whey was carefully collected and weighed, and WHC was calculated using Equation (3):

(3) $$\mathrm{WHC}\;\left(\%\right)=\left[1–\left(\text{Whey weight}/\text{initial weight}\right)\right]\times 100$$

where “whey weight” refers to the weight of the whey obtained after centrifugation and “initial weight” corresponds to the initial sample weight prior to centrifugation. WHC values were calculated based on this equation.

#### 2.7.2 Color Properties

Surface color changes of the kefir samples during storage were evaluated on the 1st, 7th, and 14th days by determining reflected color values. Color measurements were conducted using a CHN Spec. spectrophotometric colorimeter (Model CS‐410, China) based on the CIELab color space under D65 illuminant conditions with a 10° standard observer. Color properties were expressed as L*, a*, and b* coordinates, representing lightness, red–green, and yellow–blue color components, respectively (Boruczkowska et al. 2025). The overall color variation between control and fortified kefir samples was quantified by calculating the total color difference (ΔE*_ab_) according to the equation reported by De Flaviis and Sacchetti (2025). Color measurements were performed using samples obtained from two independent kefir production trials, which served as biological replicates. For each biological replicate, three color measurements were recorded at each storage time point, and the mean value was used for statistical analysis. The total color difference (ΔE*_ab_) was determined using Equation (4):

(4) $${{\mathrm{\Delta E}}^{*}}_{\mathrm{ab}}={\left[{\left({L^{*}}_{0}–L^{*}\right)}^{2}+{\left({a^{*}}_{0}–a^{*}\right)}^{2}+{\left({b^{*}}_{0}–b^{*}\right)}^{2}\right]}^{1/2}$$

where L_0_, a_0_, and b_0_ are the initial color values of the samples (Day 1), and L, a*, and b* are the corresponding color values measured during storage. L* indicates lightness, a* represents the red‐green axis, and b* represents the yellow‐blue axis.

#### 2.7.3 Total Antioxidant Activity

The antioxidant potential of the samples was assessed using the ABTS TEAC assay. The ABTS^+^ radical cation was generated by reacting a 7 mM ABTS solution with 2.45 mM potassium persulfate. Before analysis, the ABTS^+^ solution was diluted with phosphate‐buffered saline (PBS, pH 7.4) to obtain an absorbance of 0.70 ± 0.02 at 734 nm and incubated at 30°C. The radical scavenging activity of the samples in the presence of Trolox (6‐hydroxy‐2,5,7,8‐tetramethylchroman‐2‐carboxylic acid) was measured spectrophotometrically at 734 nm using a Shimadzu UV–Vis spectrophotometer (Shimadzu Scientific Instruments, Tokyo, Japan). Antioxidant capacity was quantified using the TEAC method by comparing sample responses with a Trolox calibration curve, and results were expressed as Trolox equivalents (μmol TE/mL), following Re et al. (1999).

#### 2.7.4 Total Phenolic Content Analysis

The total phenolic content of the kefir samples was evaluated using the Folin–Ciocalteu colorimetric method according to the procedure reported by Singleton and Rossi (1965). Gallic acid served as the standard compound for calibration. Absorbance readings were taken at 760 nm using a Shimadzu spectrophotometer (Shimadzu Scientific Instruments Inc., Tokyo, Japan). The phenolic content of the samples was calculated from the gallic acid calibration curve and expressed as milligrams of gallic acid equivalents (GAE) per liter of sample (mg GAE/L).

#### 2.7.5 Texture Analysis

The textural parameters of kefir samples (firmness, consistency, cohesiveness, and work of cohesion) were determined using a texture analyzer (Stable Micro Systems, TA‐XT plusC, Godalming, Surrey, UK) equipped with a 5 kg load cell, following a modified version of the method described by Glibowski and Kowalska (2012). For each measurement, 20 mL of kefir sample was placed into a plastic container (45 mm in diameter and 25.5 mm in height), resulting in a sample height of approximately 18 mm. The analysis was carried out using a cylindrical probe with a diameter of 35 mm. Measurements were performed at 25°C by compressing the samples to a depth of 10 mm at a constant speed of 1.0 mm/s. The back‐extrusion technique was applied to perform Texture Profile Analysis (TPA) using Texture Exponent software (version 8.1.9.0, Stable Micro Systems). Texture analysis was performed using samples obtained from two independent kefir production trials, which served as biological replicates. For each biological replicate, four instrumental measurements were performed, and the mean value was used for statistical analysis.

### 2.8 Statistical Analysis

Each kefir formulation was produced in two independent production trials, which served as biological replicates. Within each biological replicate, analytical measurements were performed as technical replicates according to the respective analytical method (duplicate measurements for pH, titratable acidity, total phenolic content, antioxidant activity, and water‐holding capacity; triplicate measurements for color; and four instrumental measurements for texture analysis). Statistical evaluation of the data was performed using SPSS software for Windows (version 16.0). The data were analyzed using one‐way analysis of variance (ANOVA), and the level of statistical significance was set at *p* < 0.05. When significant differences were detected, Duncan's multiple range test was applied for post hoc comparison of the means (SPSS 2017). Additionally, a post hoc power analysis was performed using G*Power software (version 3.1.9.7) based on representative quality parameters with observable variability (firmness, consistency, and ΔE*_ab_). The obtained power values ranged from 0.91 to 1.00, indicating sufficient statistical power to detect the observed effects under the current experimental conditions (Faul et al. 2009).

## 3 Results and Discussion

### 3.1 Characterization of Alginate Beads

Based on the reproducibility assessment across different batches, the average encapsulation efficiency (EE%) and loading capacity (LC%) of mastic gum extract encapsulated in alginate beads were 78.15% ± 0.6% and 15.3% ± 0.8%, respectively (*n* = 2). The total and surface phenolic contents of the encapsulated mastic gum extract were 10.39 ± 0.10 mg GAE/g dry solid and 2.27 ± 0.03 mg GAE/g dry solid, respectively, indicating successful incorporation of phenolic compounds into the alginate beads (*n* = 2). These values were used for the calculation of encapsulation efficiency and loading capacity. The encapsulation efficiency (EE%) obtained in this study (78.15% ± 0.6%) is consistent with the typical range reported for alginate‐based encapsulation systems used for plant‐derived bioactive compounds. In the literature, EE values for alginate beads generally vary between approximately 50% and 90%, depending on factors such as polymer concentration, cross‐linking conditions, and the physicochemical properties of the encapsulated compounds (Machado et al. 2022; Kučuk et al. 2024; Toprakçı et al. 2025). Phenolic‐rich plant extracts, in particular, have been reported to exhibit relatively high encapsulation efficiencies due to their ability to interact with the alginate matrix through hydrogen bonding and electrostatic interactions (Kalita et al. 2026). The EE value obtained in the present study falls within this commonly reported range, suggesting efficient entrapment of mastic gum extract within the alginate network (Li et al. 2024; Colin et al. 2024). In addition, the loading capacity (LC%) value (15.3% ± 0.8%) is comparable with previously reported values for hydrogel‐based encapsulation systems, where LC is influenced by formulation parameters such as polymer‐to‐core ratio and the physicochemical properties of the bioactive compounds (Huang et al. 2023; Chen et al. 2024; Harlen et al. 2025). The relatively low standard deviation observed for both EE and LC suggests good reproducibility of the encapsulation process under the applied experimental conditions. Overall, these results indicate that alginate‐based encapsulation can serve as a suitable approach for incorporating plant‐derived bioactive compounds into a delivery system.

The mean diameter of the produced capsules was 3.00 ± 0.20 mm (*n* = 10), measured using a digital caliper, which is within the typical size range reported for alginate‐based hydrogel beads intended for bioactive compound encapsulation. Capsule size is an important factor that may influence encapsulation efficiency and the release behavior of encapsulated compounds. Smaller beads generally provide higher surface‐area‐to‐volume ratios, which may facilitate diffusion, whereas larger beads may contain greater amounts of core material and potentially higher loading capacity (Chen et al. 2024). Similar studies have reported mean diameters ranging from approximately 2.5–4.0 mm for alginate hydrogel beads prepared via extrusion methods, highlighting the reproducibility of this fabrication approach (Aykın Dinçer et al. 2024; Morales et al. 2024; Sıçramaz et al. 2025). The relatively low standard deviation observed in the current study indicates consistent bead formation under the applied preparation conditions, suggesting that the selected extrusion parameters and polymer concentration were appropriate for producing alginate beads with uniform morphology.

### 3.2 Scanning Electron Microscopy (SEM) Analysis

**FIGURE 2:**
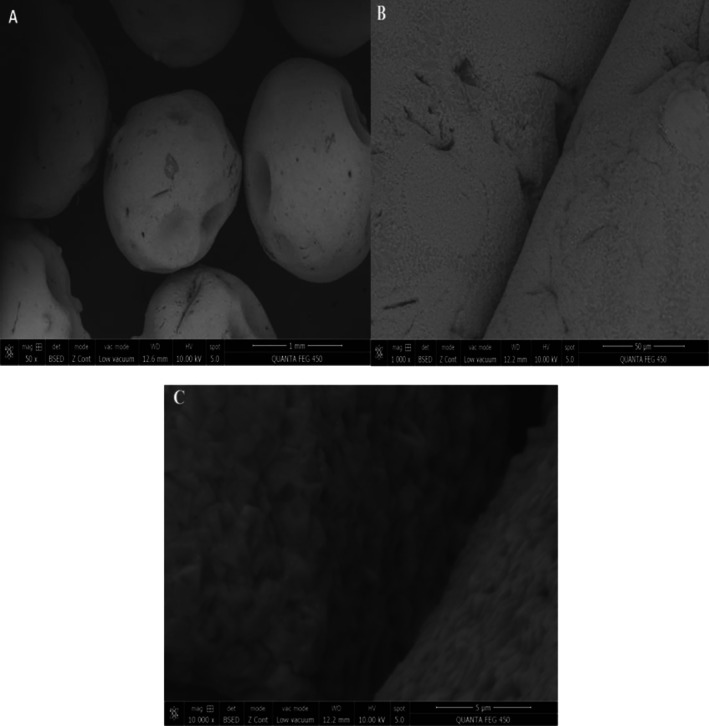
SEM micrographs of dried alginate‐encapsulated mastic gum extract beads obtained at different magnifications: (A) ×50, (B) ×1000, and (C) ×10,000. The images show the morphology, surface characteristics, and structural integrity of the alginate‐encapsulated beads after the encapsulation process.

The SEM image of the alginate‐encapsulated mastic gum beads (Figure 2) showed that the capsules were predominantly spherical in shape with relatively smooth and uniform surfaces. Minor surface irregularities were observed, which are commonly reported for alginate‐based beads produced by ionic gelation. This morphology may be attributed to the rapid cross‐linking of calcium ions at the droplet surface, resulting in the formation of a denser outer layer (Lee and Mooney 2012; Silva et al. 2014). The capsules exhibited relatively uniform morphology, with no evident agglomeration or major structural collapse. These observations suggest that the encapsulation process resulted in structurally intact beads. Additionally, the observed surface characteristics may have been partially influenced by the drying procedure applied prior to SEM analysis (Parvez and Wani 2024). The absence of visible cracks and severe structural defects indicates the potential suitability of alginate beads as carriers for mastic gum extract and their possible role in modulating the release of bioactive compounds.

### 3.3 Physicochemical Properties

The evolution of titratable acidity, pH, and water holding capacity (WHC) of kefir samples during refrigerated storage is presented in Table 2, revealing statistically significant effects of both formulation and storage time (*p* < 0.05).

**TABLE 2:** Physicochemical properties of kefir samples.

Sample	Storage periods (day)	Titratable acidity (LA%)	pH	Water holding capacity (WHC) (%)
C	1	0.76 ± 0.01^Aa^	4.69 ± 0.00^Bc^	37.36 ± 0.32^Ac^
	7	0.79 ± 0.01^Bb^	4.57 ± 0.00^Ab^	35.79 ± 0.21^Ab^
	14	0.99 ± 0.02^Bc^	4.52 ± 0.00^Aa^	34.96 ± 0.20^Aa^
FM‐1	1	0.76 ± 0.00^Aa^	4.61 ± 0.00^Ac^	40.73 ± 0.26^Ca^
	7	0.74 ± 0.01^ABa^	4.60 ± 0.00^Db^	41.56 ± 0.19^Bb^
	14	0.96 ± 0.01^Bb^	4.58 ± 0.00^Ca^	41.78 ± 0.31^Bb^
FM‐2	1	0.77 ± 0.00^Aa^	4.64 ± 0.00^Cc^	40.06 ± 0.10^ABa^
	7	0.78 ± 0.02^Ba^	4.61 ± 0.00^Cb^	42.15 ± 0.02^Bb^
	14	0.98 ± 0.01^Bb^	4.59 ± 0.00^Da^	41.40 ± 0.22^Bc^
EM‐1	1	0.77 ± 0.01^Aa^	4.67 ± 0.00^Dc^	43.67 ± 0.22^Ba^
	7	0.79 ± 0.01^Ba^	4.58 ± 0.00^Bb^	45.80 ± 0.20^Cb^
	14	0.93 ± 0.00^Ab^	4.57 ± 0.00^Ba^	47.10 ± 0.37^Dc^
EM‐2	1	0.78 ± 0.00^Aa^	4.68 ± 0.00^Bc^	42.49 ± 0.16^Da^
	7	0.79 ± 0.01^Ba^	4.59 ± 0.00^Eb^	43.70 ± 0.57^Bb^
	14	0.94 ± 0.01^Bb^	4.58 ± 0.00^Ca^	43.61 ± 0.05^Cb^

*Note:* Different lowercase letters (^a–c^) for the same treatment indicate significant differences among storage days (1, 7, and 14 days) (*p* < 0.05). Different uppercase letters (^A–E^) within the same storage day indicate significant differences among kefir formulations (*p* < 0.05).

Abbreviations: C, plain kefir as the control; EM‐1, kefir supplemented with 0.1% alginate‐encapsulated mastic gum extract; EM‐2, kefir supplemented with 0.5% alginate‐encapsulated mastic gum extract; FM‐1, kefir containing 0.1% free mastic gum extract; FM‐2, kefir containing 0.5% free mastic gum extract.

Titratable acidity increased progressively in all samples, confirming the continuation of post‐acidification during storage. However, the magnitude of this increase was formulation‐dependent. The control sample exhibited the highest increment (0.76%–0.99% LA), whereas kefir samples containing mastic gum extract showed a comparatively lower increase in acidity, with the extent of change depending on the formulation. This behavior may be associated with possible interactions between phenolic compounds and components of the kefir matrix during storage (Popescu et al. 2023). In contrast, the free extract samples (FM‐1 and FM‐2) also showed changes in acid development, suggesting that the effects of extract incorporation were not limited to the encapsulated form (Gaur and Gänzle 2023).

The decline in pH values across all treatments corroborated the accumulation of organic acids. Although extract‐containing samples generally maintained slightly higher pH values compared with the control, the magnitude of these differences varied depending on formulation and storage time (*p* < 0.05). This trend suggests that mastic gum extract may influence acidification behavior through possible interactions within the kefir matrix (Terpou et al. 2018). Such effects have been associated with phenolic‐rich plant extracts, which may interfere with enzymatic activity and microbial growth patterns (Takó et al. 2020). The differences observed between free and encapsulated formulations indicate that the effect of encapsulation on acidification was not consistent across all formulations and should be interpreted considering the specific storage conditions and formulation characteristics. WHC values varied among treatments and storage periods. Among the evaluated formulations, EM‐1 showed the highest WHC value at Day 14 (47.10%), indicating a higher water retention capacity under this specific storage condition. This behavior may be explained by multiple interacting factors. Firstly, alginate‐based encapsulation may contribute to changes in the gel structure through calcium‐mediated interactions, potentially affecting the organization of the protein–polysaccharide network (Feng et al. 2024). Secondly, interactions between milk proteins and phenolic compounds may influence water‐binding capacity by modifying the availability of hydrophilic sites within the gel structure (Martinović et al. 2024). The changes observed in WHC during storage may also be related to structural rearrangements occurring within the kefir matrix (Ramdhan et al. 2021).

From a comparative perspective, the incorporation of encapsulated and free mastic gum extract resulted in different effects depending on the evaluated physicochemical parameter. These findings indicate that increasing extract concentration did not necessarily lead to proportional improvements, suggesting that the effect of extract incorporation depends on concentration level and matrix interactions (Nieto et al. 2023). Similar work using Chios mastic gum as an encapsulating matrix for probiotic cells in fermented milk demonstrated that mastic resin can form a stable encapsulation system and influence microbial activity and product properties during storage, supporting the potential application of mastic‐derived materials in functional fermented dairy products (Terpou et al. 2018).

### 3.4 Color Characteristics

The color characteristics of all kefir samples were monitored during the storage period, and the obtained results are presented in Table 3.

**TABLE 3:** Instrumental color parameters of kefir samples.

Sample	Storage periods (day)	L*	a*	b*	ΔE*_ab_
C	1	71.82 ± 0.83^Aa^	−0.42 ± 0.12^Aa^	6.91 ± 0.23^Aa^	—
	7	78.77 ± 0.38^Bb^	−0.41 ± 0.11^Aa^	7.33 ± 0.13^Aa^	6.96 ± 0.39^Ba^
	14	79.72 ± 0.44^Bb^	−0.42 ± 0.02^Aa^	7.31 ± 0.08^Aa^	7.91 ± 0.44^Ba^
FM‐1	1	73.72 ± 0.56^Aa^	0.43 ± 0.10^Cc^	7.73 ± 0.18^Bb^	2.23 ± 0.57^Aa^
	7	72.55 ± 0.23^Aa^	−0.26 ± 0.12^Ab^	7.04 ± 0.19^Aab^	0.75 ± 0.21^Aa^
	14	79.20 ± 1.38^Bb^	−0.43 ± 0.11^Aa^	7.36 ± 0.09^Ab^	7.39 ± 1.38^Bb^
FM‐2	1	72.76 ± 0.32^Aa^	0.04 ± 0.11^Ba^	7.03 ± 0.22^Aa^	1.05 ± 0.30^Aa^
	7	71.79 ± 0.23^Aa^	0.14 ± 0.06^Ba^	7.34 ± 0.03^Aab^	0.70 ± 0.20^Aa^
	14	76.47 ± 0.25^Cb^	0.33 ± 0.08^Ba^	7.84 ± 0.15^ABb^	4.79 ± 0.26^ABb^
EM‐1	1	79.08 ± 0.55^Ba^	−0.26 ± 0.01^ABa^	7.60 ± 0.11^ABb^	7.29 ± 0.53^Bb^
	7	74.10 ± 1.45^Ab^	−0.33 ± 0.18^Aa^	6.80 ± 0.30^Aab^	2.28 ± 1.46^Aa^
	14	78.60 ± 0.98^BCa^	−0.26 ± 0.16^Aa^	7.02 ± 0.34^Ab^	6.78 ± 0.99^Bb^
EM‐2	1	71.79 ± 0.85^Aa^	−0.06 ± 0.19^ABa^	6.90 ± 0.20^Aa^	0.35 ± 0.77^Aa^
	7	73.50 ± 1.02^Aa^	−0.05 ± 0.07^ABa^	6.80 ± 0.22^Aa^	1.72 ± 1.02^Aab^
	14	74.40 ± 1.07^Aa^	0.14 ± 0.13^Bb^	7.09 ± 0.30^Aa^	2.64 ± 1.08^Ab^

*Note:* Different lowercase letters (^a–c^) for the same treatment indicate significant differences among storage days (1, 7, and 14 days) (*p* < 0.05). Different uppercase letters (^A–C^) within the same storage day indicate significant differences among kefir formulations (*p* < 0.05).

Abbreviations: C, plain kefir as the control; EM‐1, kefir supplemented with 0.1% alginate‐encapsulated mastic gum extract; EM‐2, kefir supplemented with 0.5% alginate‐encapsulated mastic gum extract; FM‐1, kefir containing 0.1% free mastic gum extract; FM‐2, kefir containing 0.5% free mastic gum extract.

The color parameters (L*, a*, b*, and ΔE*_ab_) of kefir samples were significantly influenced by both formulation and storage time (*p* < 0.05). The lightness (L*) values generally ranged between 71.79 and 79.72, indicating that all samples maintained a relatively high brightness typical of kefir. The control sample exhibited an increase in L* values during storage, suggesting minor changes in matrix structure or light scattering properties. Similar trends have been reported in fermented dairy products enriched with plant‐derived phenolics, often associated with pigment transformations during storage (Wijesekara et al. 2022). A comparable increasing trend was observed in FM‐1 and FM‐2 by day 14, whereas some encapsulated formulations, particularly EM‐2, showed relatively stable L* values over time.

The a* values remained close to zero across all samples, indicating the absence of strong red or green coloration. FM‐1 showed an initial positive a* value that decreased during storage, while other formulations exhibited minor fluctuations without a consistent trend. Changes in a* values in fermented dairy systems are generally associated with pigment stability and interactions with the food matrix, including protein–phenolic interactions (Ścibisz and Ziarno 2023). All samples displayed positive b* values, reflecting the characteristic creamy‐yellow color of kefir. FM‐2 showed an increase in b* values at Day 14, likely related to higher extract concentration, whereas other samples exhibited only slight variations during storage. Similar findings have been reported in dairy systems enriched with plant‐based extracts, where concentration influences color intensity (Keum et al. 2025). Color difference (ΔE*_ab_) values provided an overall indication of color changes during storage. Values below 1 are generally considered imperceptible, values between 1 and 3 indicate slight differences, and values above 3 correspond to visually noticeable changes (Cserhalmi et al. 2006). In this study, ΔE*_ab_ values indicated that color changes became more evident in some formulations after 14 days of storage. The control and FM‐1 samples showed higher ΔE*_ab_ values compared with some encapsulated formulations, particularly EM‐2, which showed relatively lower ΔE*_ab_ values under the evaluated storage conditions. These differences suggest that formulation type and extract concentration played a role in color stability during storage. Overall, the results indicate that mastic gum extract influenced the color properties of kefir, with variations depending on formulation type and storage time. Encapsulated formulations did not show a uniform response across all color parameters; however, some formulations exhibited lower changes depending on the specific color attribute and storage conditions.

### 3.5 Assessment of the Overall Antioxidant Activity and Phenolic Content

**FIGURE 3:**
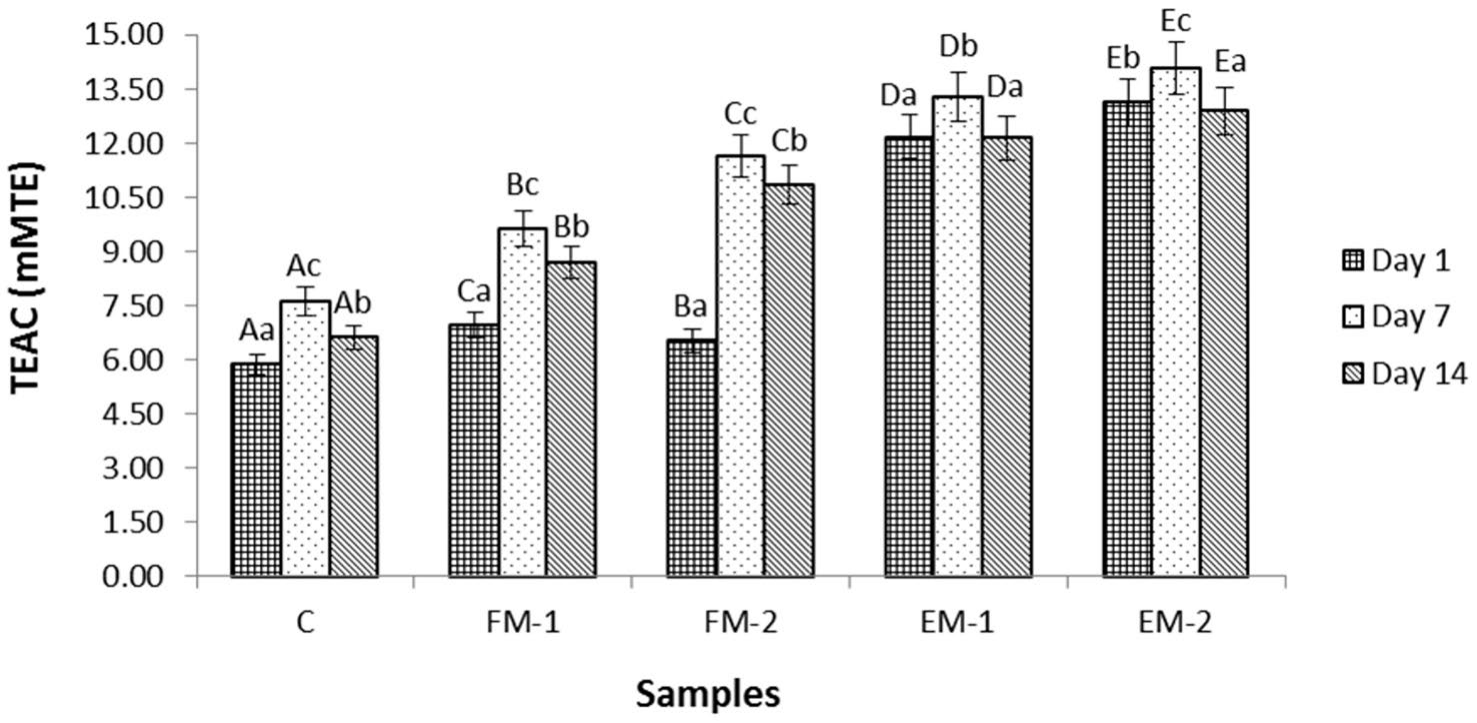
Changes in the overall antioxidant capacity of kefir samples throughout storage. C: Plain kefir as the control; FM‐1: Kefir containing 0.1% free mastic gum extract; FM‐2: Kefir containing 0.5% free mastic gum extract; EM‐1: Kefir supplemented with 0.1% alginate‐encapsulated mastic gum extract; EM‐2: Kefir supplemented with 0.5% alginate‐encapsulated mastic gum extract. Different lowercase letters (^a–c^) for the same treatment indicate significant differences among storage days (1, 7, and 14 days) (*p* < 0.05). Different uppercase letters (^A–E^) within the same storage day indicate significant differences among kefir formulations (*p* < 0.05).

The changes in the overall antioxidant capacity of kefir samples during storage are presented in Figure 3. The results demonstrated that both the formulation and storage time significantly affected the antioxidant activity of the samples (*p* < 0.05). Kefir samples supplemented with mastic gum extract exhibited higher antioxidant activity compared to the control, confirming the contribution of phenolic‐rich plant extracts to the functional properties of fermented dairy products (Kandyliari et al. 2023). Specifically, the control sample showed relatively low values, increasing from 5.87 on Day 1 to 7.61 on Day 7, followed by a slight decrease to 6.60 on Day 14. In contrast, FM‐1 increased from 6.95 to 9.63 and then slightly decreased to 8.69, while FM‐2 showed a more pronounced rise from 6.53 on Day 1 to 11.65 on Day 7, maintaining 10.85 at Day 14. This effect was more pronounced in samples containing higher concentrations of the extract (FM‐2 and EM‐2), suggesting a concentration‐related trend between extract level and antioxidant capacity.

During storage, fluctuations in antioxidant activity were observed across all groups. In free extract‐containing samples (FM‐1 and FM‐2), antioxidant capacity tended to show greater variability, which may be associated with possible interactions of phenolic compounds with the kefir matrix, as well as their susceptibility to oxidation and degradation over time. These interactions may lead to structural modifications or reduced bioavailability of active compounds (Wróblewska et al. 2023). Encapsulated samples (EM‐1 and EM‐2) exhibited higher antioxidant activity values throughout storage. However, the observed differences in temporal variation between free and encapsulated formulations should be interpreted cautiously. EM‐1 increased from 12.16 on Day 1 to 13.28 on Day 7 and remained relatively stable at 12.14 on Day 14, while EM‐2 showed the highest overall values (13.12, 14.08, and 12.88 for Days 1, 7, and 14, respectively). Compared with the corresponding free extract formulations at the same extract concentration, encapsulated samples consistently exhibited higher antioxidant activity throughout storage. This observation may be attributed to the protective effect of the alginate matrix, which can reduce the exposure of phenolic compounds to oxidation and interactions with milk proteins, thereby preserving their antioxidant potential (Barutçu Mazi et al. 2026). In addition, the gradual release of bioactive compounds from the alginate matrix may have contributed to maintaining antioxidant activity during storage (Colin et al. 2024). Nevertheless, these findings should be interpreted with caution, as the magnitude of the encapsulation effect varied depending on the storage period and the statistical differences observed among formulations. Overall, these findings suggest that alginate encapsulation may contribute to maintaining antioxidant activity during storage, although its effectiveness relative to free extract depended on the storage period and the formulation.

**FIGURE 4:**
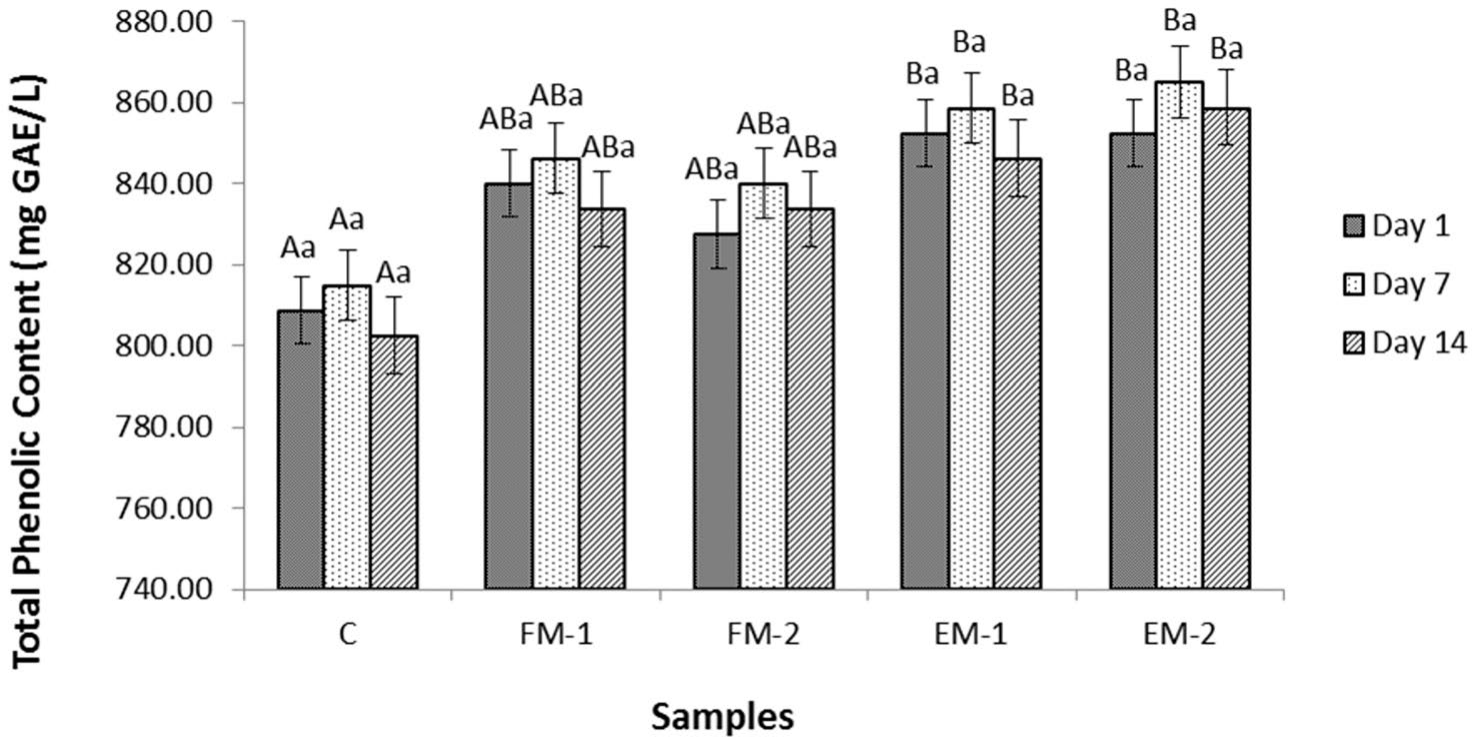
Changes in the total phenolic content of kefir samples throughout storage. C: Plain kefir as the control; FM‐1: Kefir containing 0.1% free mastic gum extract; FM‐2: Kefir containing 0.5% free mastic gum extract; EM‐1: Kefir supplemented with 0.1% alginate‐encapsulated mastic gum extract; EM‐2: Kefir supplemented with 0.5% alginate‐encapsulated mastic gum extract. Different lowercase letters (^a–b^) for the same treatment indicate significant differences among storage days (1, 7, and 14 days) (*p* < 0.05). Different uppercase letters (^A–B^) within the same storage day indicate significant differences among kefir formulations (*p* < 0.05).

The changes in total phenolic content (TPC) of kefir samples during storage are presented in Figure 4. The results indicated that both the formulation and storage time had a statistically significant effect on TPC values (*p* < 0.05). The incorporation of mastic gum extract, whether in free or encapsulated form, significantly enhanced the phenolic content compared to the control sample, confirming that mastic gum is a rich source of phenolic compounds and contributes to the functional enrichment of fermented dairy products (Paraschos et al. 2012; Terpou et al. 2018). Among the supplemented samples, TPC values differed according to formulation; however, a consistent concentration‐dependent increase was not observed across all formulations. This finding is consistent with previous studies reporting that plant‐derived extracts contribute to increased phenolic content owing to their inherent bioactive composition (Kandyliari et al. 2023; Añibarro‐Ortega et al. 2025).

Although numerical differences in TPC were observed among formulations, increasing the concentration of mastic gum extract did not consistently result in higher TPC values, and the differences between concentration levels were not always statistically significant. This observation suggests that increasing the amount of mastic gum extract did not necessarily result in a proportional increase in measurable phenolic compounds within the kefir matrix. Possible explanations include interactions between phenolic compounds and milk proteins, which may reduce the availability of phenolics for analytical determination, as well as matrix‐related effects influencing phenolic recovery.

During storage, fluctuations in TPC were observed across all samples, as indicated by the lowercase letter groupings (*p* < 0.05). These changes may be attributed to several factors, including interactions between phenolic compounds and milk proteins, oxidative degradation, and microbial metabolism during fermentation and storage (Han et al. 2019). In particular, a decrease in TPC over time in some samples could be associated with the binding of phenolic compounds to casein micelles or their transformation into other metabolites (Tosif et al. 2021; Chen et al. 2022). In this context, encapsulation may help mitigate such interactions by providing a physical barrier around bioactive compounds.

Both free and encapsulated formulations showed elevated phenolic contents compared with the control. Although encapsulated samples tended to exhibit slightly less variation during storage, statistically significant differences between free and encapsulated formulations were limited. Therefore, the potential protective effect of encapsulation on phenolic compounds should be interpreted with caution. The relative stability observed in some encapsulated samples may be associated with the barrier properties of alginate; however, this effect was not consistently reflected in statistically significant differences between free and encapsulated formulations. Moreover, the gradual release of phenolics from alginate microcapsules may influence their behavior during storage (Terpou et al. 2018; Kalita et al. 2026). The uppercase letter groupings further confirmed significant differences among sample types (*p* < 0.05), suggesting that formulation characteristics, including extract concentration and delivery form, may influence the phenolic profile of kefir. Taken together, encapsulation may contribute to the maintenance of phenolic compounds in fermented dairy systems; however, its effect relative to free extract depended on storage time and the parameter evaluated.

### 3.6 Textural Characteristics of Kefir

The texture profile analysis (TPA) parameters of kefir samples, including firmness, consistency, cohesiveness, and work of cohesion, were generally not significantly affected by formulation or storage time (*p* > 0.05) (Table 4).

**TABLE 4:** Texture profile analysis (TPA) parameters of kefir samples during storage.

Sample	Storage periods (day)	Firmness (N)	Consistency (N·s)	Cohesiveness (N)	Work of cohesion (N·s)
C	1	0.125 ± 0.001^Aa^	0.931 ± 0.013^Aa^	−0.093 ± 0.000^Aa^	−0.007 ± 0.000^Ba^
	7	0.130 ± 0.002^Aa^	0.927 ± 0.013^Aa^	−0.095 ± 0.004^Aa^	−0.006 ± 0.000^Ba^
	14	0.128 ± 0.004^Aa^	0.964 ± 0.032^Aa^	−0.095 ± 0.002^Aa^	−0.007 ± 0.000^Aa^
FM‐1	1	0.131 ± 0.001^Aa^	0.936 ± 0.002^Aa^	−0.099 ± 0.001^Aa^	−0.008 ± 0.000^Aa^
	7	0.118 ± 0.003^Aa^	0.917 ± 0.017^Aa^	−0.097 ± 0.004^Aa^	−0.008 ± 0.000^Aa^
	14	0.126 ± 0.002^Aa^	0.945 ± 0.015^Aa^	−0.096 ± 0.004^Aa^	−0.007 ± 0.000^Aab^
FM‐2	1	0.127 ± 0.003^Aa^	0.941 ± 0.007^Aa^	−0.096 ± 0.003^Aa^	−0.007 ± 0.000^Ba^
	7	0.124 ± 0.001^Aa^	0.926 ± 0.020^Aa^	−0.090 ± 0.001^Aa^	−0.006 ± 0.000^Ba^
	14	0.128 ± 0.003^Aa^	0.919 ± 0.015^Aa^	−0.095 ± 0.003^Aa^	−0.007 ± 0.000^Aab^
EM‐1	1	0.129 ± 0.002^Aa^	0.921 ± 0.009^Aa^	−0.092 ± 0.001^Aa^	−0.007 ± 0.000^Ba^
	7	0.126 ± 0.003^Aa^	0.988 ± 0.023^Bb^	−0.090 ± 0.004^Aa^	−0.006 ± 0.000^Bb^
	14	0.127 ± 0.002^Aa^	0.945 ± 0.013^Aab^	−0.093 ± 0.002^Aa^	−0.007 ± 0.000^Aab^
EM‐2	1	0.139 ± 0.008^Aab^	0.934 ± 0.017^Aa^	−0.096 ± 0.003^Aa^	−0.007 ± 0.000^Ba^
	7	0.130 ± 0.008^Aa^	0.919 ± 0.006^Aa^	−0.099 ± 0.002^Aa^	−0.007 ± 0.000^BCa^
	14	0.146 ± 0.010^Bb^	0.918 ± 0.008^Aa^	−0.096 ± 0.002^Aa^	−0.007 ± 0.000^Aa^

*Note:* Different lowercase letters (^a–b^) for the same treatment indicate significant differences among storage days (1, 7, and 14 days) (*p* < 0.05). Different uppercase letters (^A–C^) within the same storage day indicate significant differences among kefir formulations (*p* < 0.05).

Abbreviations: C, plain kefir as the control; EM‐1, kefir supplemented with 0.1% alginate‐encapsulated mastic gum extract; EM‐2, kefir supplemented with 0.5% alginate‐encapsulated mastic gum extract; FM‐1, kefir containing 0.1% free mastic gum extract; FM‐2, kefir containing 0.5% free mastic gum extract.

Firmness values of the samples ranged between 0.118 N and 0.146 N throughout storage. Overall, no pronounced fluctuations were observed in the control and most treatment groups, indicating that the kefir matrix maintained its structural integrity during storage. However, the EM‐2 sample exhibited a significant increase in firmness at Day 14 (*p* < 0.05). This isolated change suggests that formulation‐specific interactions may influence textural behavior under certain storage conditions. Such differences may be related to changes in matrix organization during storage; however, further studies are needed to clarify the underlying mechanisms.

Consistency values ranged from 0.917 N to 0.988 N, indicating generally stable consistency across the samples during storage. However, EM‐1 exhibited a significant increase at Day 7, followed by a slight decrease at day 14 (*p* < 0.05). This transient increase may be associated with temporary structural changes within the kefir matrix during storage (Wang et al. 2024). The occurrence of this change only in EM‐1 may be related to concentration‐dependent interactions between the encapsulated extract and the kefir matrix. The lower extract concentration in EM‐1 may have resulted in different protein–polysaccharide interactions during storage compared with EM‐2. However, this interpretation should be considered as a possible explanation rather than a confirmed mechanism. In contrast, free extract‐containing samples (FM‐1 and FM‐2) did not exhibit notable changes, suggesting a more limited impact on the viscosity‐related properties of kefir.

Cohesiveness values remained relatively stable across all samples, ranging from −0.090 N to −0.099 N, with no statistically significant differences observed either among treatments or during storage (*p* > 0.05). This indicates that neither free nor encapsulated mastic gum extract substantially affected the internal bonding forces of the kefir matrix. Similarly, the work of cohesion values showed minimal variation, generally ranging from −0.006 N to −0.008 N. Although minor statistical differences were detected in some samples, these changes were not consistent or systematic, suggesting that the overall structural resilience of the kefir gels was preserved.

Taken together, textural parameters remained relatively stable during storage, with only limited significant differences observed in firmness and consistency among specific encapsulated formulations. The relative stability of textural parameters may indicate that encapsulation did not negatively affect the structural properties of kefir during storage. In contrast, free extracts may interact more rapidly with milk components, potentially affecting their influence on the structural properties of the product (El‐Sayed et al. 2024). Overall, these results indicate that the incorporation of mastic gum extract, either in free or encapsulated form, does not negatively affect the textural quality of kefir. Under the evaluated storage conditions, encapsulated formulations maintained comparable textural properties without causing detrimental changes in kefir texture. These findings are in agreement with previous studies suggesting that encapsulation techniques can support the technological functionality of plant‐derived bioactive compounds in fermented dairy systems (Szpicer et al. 2025; Obayomi et al. 2026).

Although the study was based on two independent production trials, the number of biological replicates remained limited. Nevertheless, the post hoc power analysis performed for representative parameters (firmness, consistency, and ΔE*_ab_) demonstrated adequate statistical power for detecting the observed differences. However, further studies including a greater number of independent production trials are recommended to confirm these findings.

## 4 Conclusion

The present study demonstrated that alginate‐based encapsulation can be used as a feasible approach for incorporating *Pistacia lentiscus* extract into kefir. Both free and encapsulated extracts enhanced the antioxidant capacity and phenolic characteristics of kefir compared with the control. Color properties were influenced by formulation and storage time, and encapsulated formulations generally exhibited lower variations in some color parameters during storage. Although encapsulated formulations contributed to the stability of selected physicochemical and textural parameters during storage, the advantages of encapsulation compared with free extract were limited and varied depending on the evaluated parameter and storage period. Therefore, alginate encapsulation may represent a promising strategy for the development of functional kefir products enriched with plant‐derived bioactive compounds. Further studies with a higher number of replicates are needed to confirm these findings.

## Author Contributions

**Özge Duygu Okur:** conceptualization, investigation, funding acquisition, writing – original draft, writing – review and editing, visualization, validation, methodology, software, formal analysis, project administration, data curation, supervision, resources.

## Funding

The author has nothing to report.

## Ethics Statement

The author has nothing to report.

## Conflicts of Interest

The author declares no conflicts of interest.

## Data Availability

The data collected or analyzed during the study are all available in this published article.
